# Ensemble analyses improve signatures of tumour hypoxia and reveal inter-platform differences

**DOI:** 10.1186/1471-2105-15-170

**Published:** 2014-06-06

**Authors:** Natalie S Fox, Maud HW Starmans, Syed Haider, Philippe Lambin, Paul C Boutros

**Affiliations:** 1 Informatics and Bio-computing Platform, Ontario Institute for Cancer Research, Toronto, Canada; 2 Department of Radiation Oncology (Maastro), GROW-School for Oncology and Developmental Biology, Maastricht University Medical Center, Maastricht, The Netherlands; 3 Centre for Molecular Oncology, Barts Cancer Institute, London EC1M 6BQ, UK; 4 Department of Medical Biophysics, University of Toronto, Toronto, ON, Canada; 5 Department of Pharmacology and Toxicology, University of Toronto, Toronto, ON, Canada

## Abstract

**Background:**

The reproducibility of transcriptomic biomarkers across datasets remains poor, limiting clinical application. We and others have suggested that this is in-part caused by differential error-structure between datasets, and their incomplete removal by pre-processing algorithms.

**Methods:**

To test this hypothesis, we systematically assessed the effects of pre-processing on biomarker classification using 24 different pre-processing methods and 15 distinct signatures of tumour hypoxia in 10 datasets (2,143 patients).

**Results:**

We confirm strong pre-processing effects for all datasets and signatures, and find that these differ between microarray versions. Importantly, exploiting different pre-processing techniques in an ensemble technique improved classification for a majority of signatures.

**Conclusions:**

Assessing biomarkers using an ensemble of pre-processing techniques shows clear value across multiple diseases, datasets and biomarkers. Importantly, ensemble classification improves biomarkers with initially good results but does not result in spuriously improved performance for poor biomarkers. While further research is required, this approach has the potential to become a standard for transcriptomic biomarkers.

## Background

Optimizing cancer treatment aims for a cure which kills all cancerous cells in the body with as little detriment to the patient as possible. Cancer is a highly heterogeneous disease with extreme genomic, intra- and inter-tumour heterogeneity; unsurprisingly, patients show a large variety in response to treatment [1-3]. Personalizing treatment is therefore expected to improve treatment response, and thus patient outcome. For example, in some cases surgical resection of the tumour is curative; additional treatment, which has serious side-effects, is unnecessary. In contrast, other patients presenting with similar clinical characteristics (*e.g.* age, tumour site, stage and histology) could have more aggressive disease, for which adjuvant treatment is required to cure or control disease [4]. Without markers to distinguish these patients, all are given the same treatment, resulting in over-treatment in some patients and under-treatment in others.

To address this urgent clinical need, many groups have sought to create transcriptomic biomarkers using microarray-, PCR- or RNA-Seq-based assessments of mRNA abundances. The resulting multi-gene prognostic biomarkers (sometimes called signatures) can identify patient subgroups that would be particularly likely to derive benefit from more intense therapy [5,6]. However, there have been numerous challenges in the development of clinically-useful biomarkers; most published biomarkers fail to enter routine clinical practice [7].

In cancer, where heterogeneity plays such an important role, these challenges are magnified; important tumour biomarkers may be missed when using the common practice of a single tumour biopsy to direct treatment. If faced with uncertainty in biomarkers, these are deemed unsuitable for clinical applications and clinicians prefer to treat without the information and save costs [8]. In order to advance personalized medicine, robust, reproducible biomarkers are required.

We have shown that, at least in lung cancer one of the major sources of biomarker irreproducibility is their sensitivity to relatively subtle changes in pre-processing [9]. We found that analyzing a single biomarker with different pre-processing techniques yielded highly-divergent results, and these could indeed change clinical management for individual treatments [9]. However, we also found tantalizing hints that different ways of analyzing a single biomarker could be integrated: an “ensemble” of pre-processing methodologies out-performed any individual one in a 442-patient cohort of non-small cell lung cancer patients. It appears that each pre-processing technique removes a different aspect of the underlying noise in a dataset, and thus a large enough collection of them provides a more accurate estimate of the underlying biological signal.

To generalize and extend this finding, we explored the impact of data pre-processing on a micro-environmental biomarker problem: the prediction of tumour hypoxia. Tumor hypoxia (poor oxygenation) contributes to both inter- and intra-tumour heterogeneity, and can compromise cancer treatment. It is a result of the uncontrolled growth of tumour cells and the formation of an abnormal tumour vascular network [10], and is related to chemotherapy and radiotherapy resistance, tumour aggressiveness and metastasis [11]. Hypoxia is associated with poor prognosis [11], and a marker for hypoxia both identify patients with more aggressive disease and those who might benefit from specific therapeutic options [12]. Many different predictors of hypoxia have been generated [13-20]. To understand pre-processing sensitivity and how ensemble-classification can be best exploited, we evaluate this approach for 15 separate biomarkers in 10 datasets comprising transcriptomic profiles of 2,143 primary, treatment-naïve breast cancers.

## Methods

### Datasets

The ensemble approach [9] was applied to two separate groups of primary breast cancer datasets. The first group comprises 8 datasets profiled on the Affymetrix Human Genome U133A microarrays (HG-U133A), with 1,564 total patients [21-28]. The second group is made up of 2 datasets profiled on Affymetrix Human Genome U133 Plus 2.0 GeneChip Array (HG-U133 Plus 2.0), comprising a combined 579 patients [29,30]. Only datasets reflected similar disease states and profiles were included, for example datasets of metastatic tumours were excluded [31]. All samples included were treatment-naïve.

### Biomarkers

A series of 15 published hypoxia gene biomarkers were evaluated. The following signatures were included: Buffa metagene [13], Chi signature [14], Elvidge up gene set [15], Hu signature [16], the 0% and 2% early Seigneuric signatures [17], Sorensen gene set [18], Winter metagene [19] and Starmans clusters 1 to 7 [20]. Descriptions of each biomarker are given in Additional file 1: Table S1 and Additional file 2: Table S2. The signatures evaluated here only contain up-regulated genes for which high gene expression is associated with poor survival.

### Pre-processing

**Figure 1:**
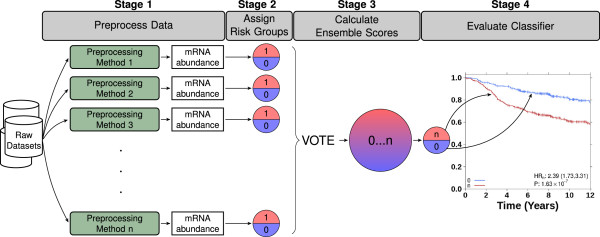
**Experimental design.** Outline of the experimental design for ensemble classification and evaluation of a biomarker. Microarray data is pre-processed in 24 different ways to calculate mRNA abundance levels (Stage 1). Risk groups are subsequently assigned for the evaluated biomarker (Stage 2). Each of the resulting classifications represents a vote for whether the patient is in the low or the high risk group. The ensemble score is a summation over these individual classifications and ranges from 0 to 24 (Stage 3). Only unanimously classified patients (ensemble scores 0 and 24) are considered robust and are evaluated with Cox proportional hazard ratio modeling and Kaplan-Meier survival curves (Stage 4).

All analyses were performed in the R statistical environment (v2.15.2). The first step was to pre-process each dataset in 24 different ways: all combinations of 6 pre-processing algorithms, 2 types of gene annotations and 2 approaches for dataset handling. Thus, each pipeline was defined by three factors (Figure 1). Each of these is outlined in detail in the following paragraphs.

The first factor creating pipeline variation for the ensemble classifier was the pre-processing algorithm. We used Robust Multi-array Average (RMA) [32], MicroArray Suite 5.0 (MAS5) [33], Model-base Expression Index (MBEI) [34], GeneChip Robust Multi-array Average (GCRMA) [35]. All of which are available in the R statistical environment (R packages: affy v1.36.0, gcrma v2.30.0). RMA and GCRMA return data in log_2_-transformed space whereas MAS5 and MBEI return data in normal space. It is common practice to log_2_-transform MAS5 and MBEI pre-processed data, therefore both normal-space and log_2_-transformed versions of these two methods were included, giving us six pre-processing algorithms.

The second factor was the annotation approach. A key part of microarray pre-processing involves mapping the 25 base-pair oligonucleotide probes to specific parts of the transcriptome (either unique transcript isoforms or full genes). This is accomplished using a chip description file (CDF). Our understanding of the human transcriptome is continually evolving, causing the annotation of individual ProbeSets to change. These advances are reflected in updated ProbeSet annotation (*i.e.* in updated CDF files) [36]. Therefore, we included both the “default" annotation (R packages: hgu133aprobe v2.10.0, hgu133acdf v2.10.0, hgu133a.db v2.8.0, hgu133plus2probe v2.6.0, hgu133plus2cdf v2.6.0, hgu133plus2.db v2.8.0) and updated Entrez Gene-based “alternative” annotation (R packages: hgu133ahsentrezgprobe v15.1.0, hgu133ahsentrezgcdf v15.0.0, hgu133plus2hsentrezgprobe v15.1.0, hgu133plus2hsentrezgcdf v15.1.0). The number of ProbeSets for each annotation is given in Table 1.

**Table 1:** Number of probe sets after pre-processing

**Microarray platform/Dataset**	**Annotation**	**Number of probe sets**
HG-U133A	default	22,283
HG-U133A	alternative	12,080
HG-U133 Plus 2.0	default	54,675
HG-U133 Plus 2.0	alternative	18,988

The number of probe sets for each annotation and microarray platform after completion of pre-processing.

The last aspect of pipeline variation considered was dataset handling. Pre-processing was either done on each dataset individually or on all datasets merged into one. Separate dataset handling involves pre-processing of a single dataset as a unit, independent of others. Each separate dataset went through the pipeline and was classified independent of the other datasets. From all separate datasets, patients classified as having good prognosis were pooled and patients predicted to have poor prognosis were pooled. Alternatively, for merged data handling, the CEL files from all datasets were combined during pre-processing and went through the entire pipeline as one dataset.

### Univariate gene analysis

For each gene represented on both array platforms, patients were median dichotomized into low and high risk groups based on the signal-intensity of that gene across all patients for a single pipeline variant. Cox proportional hazards modeling was used to assess whether survival properties were significantly different between the low risk and high risk patients. Statistical significance was assessed using the Wald test (R package: survival v2.36-14) and p-values were false-discovery rate (FDR) adjusted to correct for multiple-testing.

### Linear modeling

A simple linear model of platform, pre-processing algorithm, annotation method and dataset-handling type:

(1) $$Y=V+W+X+\sum_{i=1}^{5}Z_{i}$$

where Y is the number of genes, V is the annotation method, W is the platform, X is the data handling and Z is the pre-processing algorithm, was evaluated to determine if the model was a good fit for the data.

Second, starting with a complete model of all pairwise interactions and main effects:

(2) $$Y=V+W+X+\sum_{i=1}^{5}Z_{i}+V:W+V:X+W:X+\sum_{i=1}^{5}\left(V:Z_{i}+W:Z_{i}+X:Z_{i}\right)$$

where Y is the number of genes, V is the annotation method, W is the platform, X is the data handling and Z_1_..Z_5_ specify the 6 options for the pre-processing algorithm, backwards stepwise refinement was performed using the Akaike information criterion (AIC).

The linear modelling was constructed with alternative annotation as the baseline for V, HG-U133A as the baseline for W, merged data handling as the baseline for X, and GCRMA as the baseline for Z.

### Patient risk group classification

Each gene signature was used to classify patients into one of two groups. The number of genes present on each array for each annotation is shown in Additional file 2: Table S2. After data pre-processing, a multi-gene signature score was calculated for each patient using all genes on that platform that are in the signature's gene list:

(3) $$\mathrm{Score}=\sum_{n=1}^{N}\mathrm{gen}e_{\mathrm{expr},n}$$

where N is the number of genes in a signature and gene_expr,n_ is the median dichotomized value for the gene expression of the n^th^ gene in the signature compared to the expression levels of that gene from all samples. If the level of the n^th^ gene is above the median for all samples then gene_expr,n_ is 1, otherwise −1.

After calculating a score for each patient, these scores were used to median dichotomize patients into high and low risk groups for each signature.

### Ensemble classification

The patient risk group classifications across all pre-processing methods were combined to create an ensemble classification by looking for unanimous agreement between all pipeline variants. The high risk classification for the ensemble classification is given to the patients who have been classified as high risk in all 24 pre-processing pipeline variants; similarly for the low risk grouping. Patients with conflicting classifications between pipeline variants were deemed to have unreliable molecular classifications and were thus excluded from ensemble classification as before [9] as a conservative approach that might be used in the clinic.

### Individual classification for subset of patients

For better comparison between the ensemble classification and individual classifications, the number of patients classified based on one pre-processing approach was reduced to match the number of patients classified in the ensemble classifier. Instead of median dichotomization, the patients were ordered by their multi-gene signature score. Then the number of patients that the ensemble had classified as high risk was selected from the top of the order as high risk patients and this was equivalently done for the low risk classifications.

### Classifier evaluation

Kaplan-Meier survival curves and unadjusted Cox proportional hazard ratio modeling (R survival package, v2.36-14) were used to assess survival differences between the low risk and high risk groups. The Wald test was used to determine whether the hazard ratio was statistically different from unity. In all analyses, the superior classification was defined as the classification with the higher Cox proportional hazard ratio.

### Permutation sampling for variable number of pipelines in the ensemble

In these analyses, the ensemble classification is generally a combination of all 24 pipeline variants. However, we also varied the number of pipeline variants being combined. To represent a combination of n pipeline variants, we randomly sampled n pipelines (without replacement) and created an ensemble classifier as outlined above. This process was repeated with replacement 2000 times for each value of n ranging from 1 to 24.

### Student's t-test methods comparison

The pool of all 24 individual methods across the 15 signatures was split based on a single aspect of the pipeline (dataset handling, gene annotations or pre-processing algorithms). We compared pipelines only differing on a single aspect using the paired t-test to assess statistical differences between pipelines.

### Permutation sampling for variable number of pipelines in the ensemble when subgrouping for methods comparison

As part of the method comparison, the pipelines where subgrouped based on a single aspect of the pipeline and then within the subgroups ensembles of a varying number of the pipelines were constructed. To represent a combination of n pipeline variants, we sampled n pipelines (without replacement) and created an ensemble classifier. For each value of n (from 1 to 4 for the pre-processing algorithm or 1 to 12 if subgrouping based on gene annotation or data handling), all possible combinations containing n unique pipeline variants were created.

### Visualization

All plotting was performed in the R statistical environment (v2.15.2) using the lattice (v0.20-10), latticeExtra (v0.6-24), RColorBrewer (v1.0-5) and cluster (v1.14.3) packages.

## Results

### Ensemble classification approach

Each dataset was pre-processed using 24 different pipeline variants. Each biomarker was then applied separately for each pipeline variant, producing an ensemble of 24 predictions for each patient and biomarker. These were analyzed for consistency and combined to form a single ensemble classification. Figure 1 outlines the approach used. We separated our datasets according to the microarray platform used, and tested the two most widely-used platforms at the time of writing according to depositions in the Gene Expression Omnibus: HG-U133A and HG-U133 Plus 2.0. Since both platforms are Affymetrix arrays and therefore have the same set of potential normalization methods, we can perform inter-platform analysis independent of pre-processing.

### Univariate gene analysis

**Figure 2:**
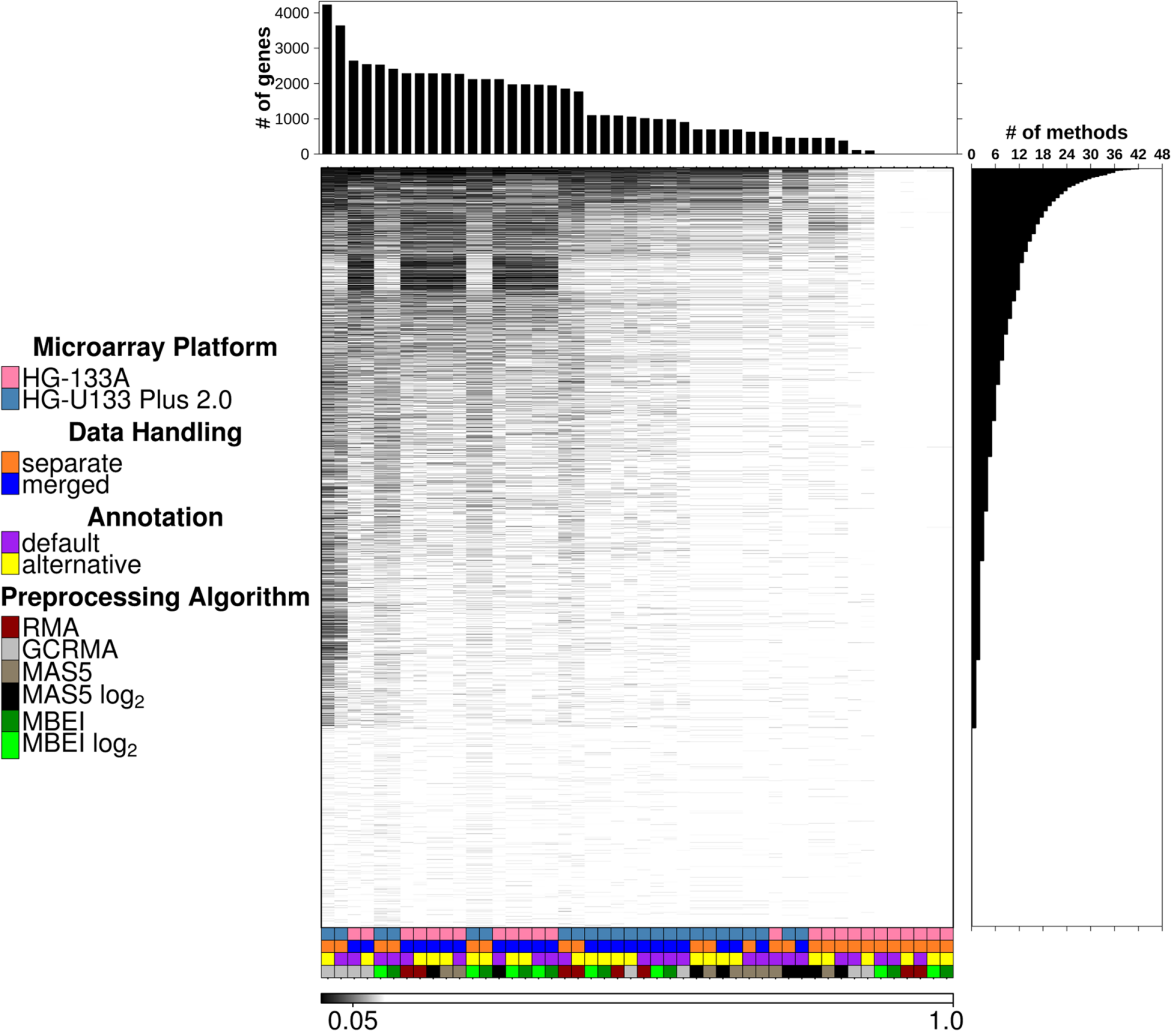
**Gene univariate analysis.** FDR-adjusted p-values (q-values) for univariate Cox proportional hazard ratio modeling analysis of all genes in common to both platforms and annotation types were visualized in a heatmap. Genes are presented along the y-axis and pipeline variants along the x-axis. The pipeline variants are specified by the covariant bar. The number of significant genes (q ≤ 0.05), per pre-processing method are provided in the top panel and the number of pre-processing methods in which each gene reaches significance (q ≤ 0.05) are displayed in the right panel.

We first investigated the univariate performance of individual genes to determine how the prognostic power of these simple biomarkers is influenced by pre-processing differences. As shown previously for lung cancer [12], the prognostic ability of individual genes varied dramatically across methods. Of the 17,701 genes represented on both array platforms tested, 74% reached statistical significance after multiple-testing correction in at least 1/24 pipeline variants. By contrast, only 16% reached significance in at least 12/24 pipelines (Figure 2) and none were significant in all pipelines. Three pipeline variants identified zero genes, while three others found a single gene (RACGAP1; Rac GTPase activating protein 1), which was not identified in the other 21 pipelines. These data clearly indicate that simple union (which would identify 74% of all genes) and intersection (no genes) approaches are inappropriate.

Interestingly, all six pipelines that resulted in either one or no prognostic genes involved analysis of HG-U133A data (n = 1,564 patients), using either the RMA or MBEI algorithms, along with the “separate” dataset-handling approach. There is an evident difference between the patterns of significant genes on each platform. The lowest concordance between pipelines is shown in the inter-platform correlations. Different aspects of the pipeline appear more highly correlated depending on the platform and there is no clear ordering of which aspect is more important without interactions (Additional file 3: Figure S1). We were able to use linear-modeling to show that the choice of pre-processing method is strongly deterministic for the number of statistically-significant genes identified. We considered a complete model of all pairwise interactions and main effects, then used the Akaike information criterion (AIC) for backwards stepwise refinement. A model containing the main effects: platform, pre-processing algorithm, data-handling type and their pairwise interactions resulted (R^2^ = 0.84; Table 2), indicating that the relationship is deterministic, not stochastic. We note that interactions are critical: a simple model of main-effects was not explanatory (R^2^ = −4.51 x 10^−3^).

**Table 2:** Significant coefficients of linear model for prognostics based on individual gene

**Coefficient**	**Estimate**	**Standard error**	**t value**	**Pr (> | t | )**
(Intercept)	1995.2	251.9	7.736	1.57×10^−8^
Handling, separate	−1305.8	313.1	−4.171	2.51×10^−4^
Platform, HGU133 Plus 2.0: Handling, separate	3079.2	236.7	13.010	1.24×10^−13^
Platform, HGU133 Plus 2.0: Algorithm, log_2_ MAS5	−1844.8	409.9	−4.500	1.02×10^−4^
Platform, HGU133 Plus 2.0: Algorithm, MAS5	−1822.2	409.9	−4.445	1.18×10^−4^
Handling, separate: Algorithm, log_2_ MAS5	−1124.2	409.9	−2.743	1.03×10^−2^
Handling, separate: Algorithm, MAS5	−1132.8	409.9	−1.461	9.83×10^−3^
Handling, separate: Algorithm, RMA	−993.0	409.9	−2.422	2.18×10^−2^

For the linear model, $Y=W+X+\sum_{i=1}^{5}Z_{i}+W:X+\sum_{i=1}^{5}\left(W:Z_{i}+X:Z_{i}\right)$ where Y is the number of genes, W is the platform, X is the data handling and Z_1_…Z_5_ are specify the 6 options for the pre-processing algorithm, the coefficients that have a p < 0.05 are shown.

### Multi-gene signatures

**Figure 3:**
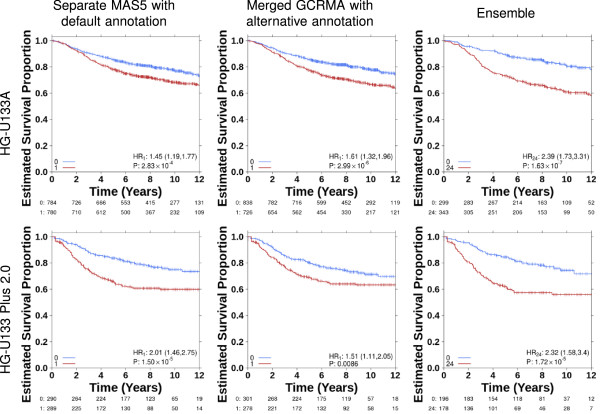
**Ensemble approach prognostic improvements.** Prognostic ability of the Winter metagene was evaluated in two breast cancer meta-datasets representing two different array platforms with Kaplan-Meier survival analyses. Two different current practice pre-processing pipelines and the ensemble approach are shown. Hazard ratios and p-values are from Cox proportional hazard ratio modeling.

**Figure 4:**
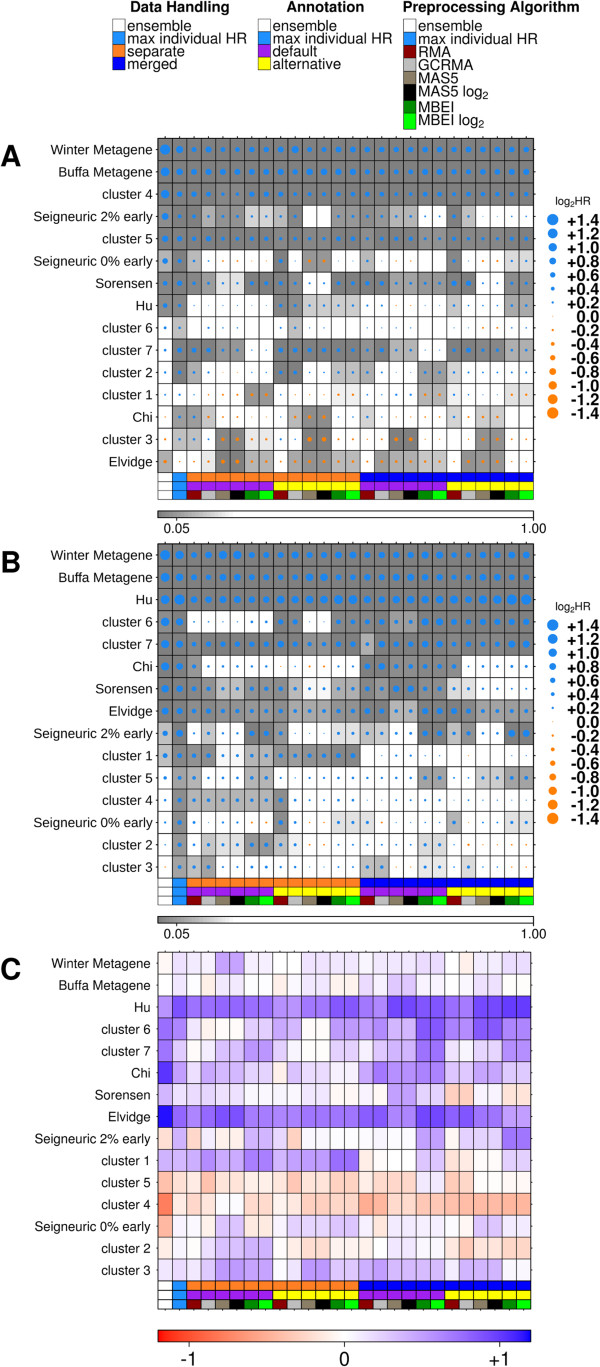
**Risk stratification across classification pipelines and prognostic signatures.** Comparison of all hazard ratios (measure of risk stratification) and corresponding p-values from Cox proportional hazard ratio modeling on **(A)** HG-U133A platform, **(B)** HG-U133 Plus 2.0 platform. The hazard ratio is represented by the size and colour of the dot and the background shade represents the p-value. Further the difference between hazard ratios on HG-U133A and HG-U133 Plus 2.0 were visualized **(C)**. A positive value (blue) represents higher log_2_ hazard ratios in HG-U133 Plus 2.0 and a negative value (red) represents higher in HG-U133A.

We next focused on multi-gene classifiers, seeking to determine if our single-gene results could be generalized. We compared the hazard ratios from Cox modeling of the ensemble and the 24 individual classifications for 15 published hypoxia signatures. For all multi-gene signatures, superior classification was defined as the classification with a higher hazard ratio. As seen with the single gene classifiers, variation was observed between classifications from the different pipelines and there was not one single variant which consistently resulted in larger risk stratification than the others. Further this analysis identified microarray platform as another possible source for variation. One pipeline variant (separate data handling, MAS5 algorithm and default annotation) showed the lowest risk stratification of the 24 pipelines on one platform (HG-U133A) and the largest of the 24 pipelines on the other platform (HG-U133 Plus 2.0) (Figure 3). As shown in Figure 3, ensemble classification performed better than individual pipelines and improved signature performance for both microarray platforms.Analyses for all signatures showed that performance was sensitive to pre-processing choices and, in the majority of cases, the ensemble classification improved prognostic ability over individual pipeline variants (Figure 4A,B). For half of the signatures, ensemble classification resulted in superior risk stratification (as measured by the magnitude of the HR) compared to classifications from the individual pre-processing pipelines. Moreover the ensemble technique was almost always superior to the “typical” pre-processing techniques, exceeding the median of the 24 techniques in 24/30 signature comparisons.

The Buffa metagene and the Winter metagene showed similar results across pipeline variants, but many of the signatures performed very differently depending on the dataset platform (Figure 4C, Additional file 4: Figure S2, Additional file 5: Table S3). Overall signatures showed better risk-stratification on HG-U133 Plus 2.0 arrays (p = 2.75 × 10^−48^, paired t-test), although this was signature-specific. Some signatures (Hu signature, Elvidge signature and Starmans cluster 3) showed consistently better results on the HG-U133 Plus 2.0 dataset compared to the HG-U133A dataset. Conversely, Starmans cluster 4 and cluster 5 performed better in the HG-U133A datasets.

The Buffa and the Winter metagene were the only signatures which were statistically significant across all pipelines tested. Hu and Sorensen, additionally, were other signatures with statistically significant ensemble classifications for both datasets. In contrast, Starmans clusters 1, 2, 3 and Seigneuric 0% early signatures did not perform well in either dataset; none of their ensemble classifications were statistically significant. In general, if a signature performed poorly for single pipeline variants, using the ensemble classification did not improve it. This was demonstrated by the correlation between the hazard ratios for the ensemble classification and the maximum hazard ratios for classification from the individual pipeline variants (R = 0.87 for HG-U133A and R = 0.88 for HG-U133 Plus 2.0).

Since previous analyses involved comparing unequal numbers of patients classified, we also compared ensemble classification to classification for the individual pre-processing methods. In this way, we match patient numbers between the two conditions, removing this potential confounding variable. In general, this approach yielded fewer statistically significant results (Additional file 6: Figure S3), although both the range and the variance of hazard ratios increased for every signature using this classification algorithm. However the comparison between of ensemble classifications and individual classifications shows that patient-number differences are not the origin of the superior performance of ensemble classification. For 13/30 signatures, the ensemble classification was superior to all classifications from the individual pre-processing pipelines and in 26/30 signatures the ensemble exceeded the median classification.

### Signature comparison

**Figure 5:**
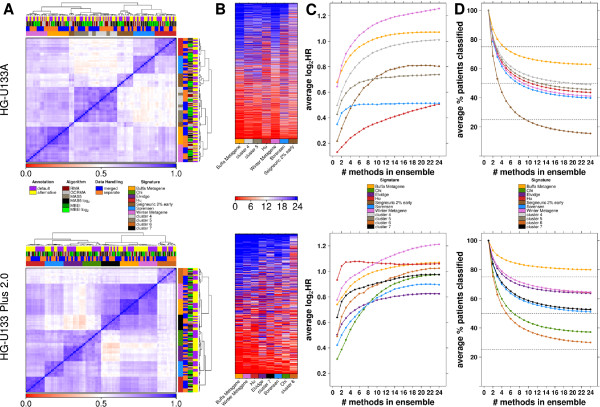
**Signature comparison.** Analysis of consistency between signatures. In **A**, heatmaps are shown for the pair-wise comparison of all the individual pipeline variants. The pipelines are compared using the percent agreement between the patient grouping for the two pipelines. **B**, shows the ensemble scores (range 0 to 24) per patient for each signature, patients are on the y-axis and signatures on the x-axis. The signatures are ordered by the number of patients classified unanimously; the signature which was most consistent across single pipeline classifications is on the far left and the least consistent one is on the right. Finally, the scatter plots compare all significant signatures when the number of pipelines used to create the ensemble classification is varied. In **C**, each point is the log_2_ of the mean hazard ratio of 2000 permutations. **D**, similarly shows the effect of the number of methods combined on the number of patients classified. For each array platform, only the signatures which have statistically significant prognostic power with the ensemble classifier (including all 24 methods) by Cox modeling are shown. For HG-U133 Plus 2.0, the Hu signature and the Winter Metagene signature have equivalent numbers of patients classified, therefore the Winter Metagene signature line is hiding the Hu signature.

To better understand which signatures were more successful, all individual classifications were compared. Unsupervised clustering of the percentage agreement of concordant patient classifications between individual pipeline variants for each signature showed that they mainly clustered by signature, rather than by pipeline composition (Figure 5A). This indicated that, although pre-processing substantially influenced biomarker performance, the genes in the signature characterized the overall partition and determined whether it was a poor or good biomarker. The Buffa metagene had the most consistent patient classifications across pipelines, but hazard ratios still ranged from 1.51 to 1.87. Although, we evaluated only hypoxia signatures, patient classifications did not agree across signatures (Figure 5A,B and Additional file 7: Figure S4). Signatures of ensemble classifications that were statistically significant generally classified a larger fraction of patients (Additional file 7: Figure S4B).

### What is the optimal ensemble size?

Having shown that the ensemble-approach improved classification for most biomarkers and datasets, we explored the limits of its performance. We wondered if 24 distinct pipelines were always necessary, and therefore evaluated the number of pipeline variants required for optimal performance (maximum risk stratification, as measured by the hazard ratio) of the ensemble classifier. If creating an ensemble of four pipeline variants is equally successful to one from eight variants, then it is not beneficial to introduce the complexity and computational-costs of pre-processing with four extra pipelines.

Focusing on signatures with a significant 24-pipeline ensemble, different combinations of pipelines, ranging from combinations of only 2 to all 24, were evaluated. These analyses indicated that in general increasing the number of pipeline variants resulted in an increase in absolute effect size which started to plateau as the number of methods in the ensemble increased (Figure 5C). In parallel, the percentage of patients classified with the ensemble method decreased and plateaued (Figure 5D). Most signatures shared the same shape but with different rates of hazard ratio increase. The Sorensen signature on the HG-U133A dataset plateaued at about four pipeline variants. Therefore, in this case, randomly choosing four pipeline variants to combine provided roughly the same risk stratification as using all 24 pipelines. Conversely, for the Winter metagene signature in either dataset, the mean hazard ratio continued to increase all the way up to 24 pipelines, though the curve was steeper at the beginning then in the end. Although the hazard ratio stopped increasing in some cases, stability continued to increase as the number of methods in the ensemble increased. This is demonstrated in Additional file 8: Figure S5 by the tightening of the hazard ratio range as the number of pipelines is increased.

Considering the Winter metagene signature in HG-U133A data, the ensembles created from nine or more of the 24 pipelines outperformed all single pipeline classifiers (Additional file 8: Figure S5 and Additional file 9: Table S4). Many ensembles did not require all 24 variants to be an improvement over all non-ensemble methods (Additional file 8: Figure S5, Additional file 9: Table S4, Additional file 10: Table S5). Even if the ensemble of 24 variants was not an improvement over non-ensemble methods, there may still have been an ensemble of a subset of the variants which was superior to the non-ensemble methods (Additional file 8: Figure S5, Additional file 9: Table S4, Additional file 10: Table S5). These data provide a compelling rationale to consider and evaluate ensemble pipelines for all microarray-based biomarkers.

### Methods comparison

After showing that ensembles are beneficial, we wanted to look at whether we can determine the combination of pipelines that lead to higher hazard ratios in order to add the most benefit for each additional pre-processing pipeline. There is a clear relationship between the number of patients classified in the ensemble and the gain in hazard ratio, meaning that the ensemble is choosing to exclude the right subset of patients (Additional file 11: Figure S6A). Methods that produce less-correlated classifications gain more from the ensemble classification. However, if we look at which methods are diverse by a different metric such as the profiles of prognostic ability of each gene as a single gene classifier, there is only a slight but not obvious increase in hazard ratio from using more diverse pipelines in the ensemble classification (Additional file 11: Figure S6B).

To help direct pipeline choices, we sought to address whether certain aspects of the pipeline resulted in better or worse performance. For each aspect of the pipeline (dataset handling, gene annotations, and pre-processing algorithms), the hazard ratios were grouped per variant of that aspect and compared. This was done for both platforms separately and combined.

**Figure 6:**
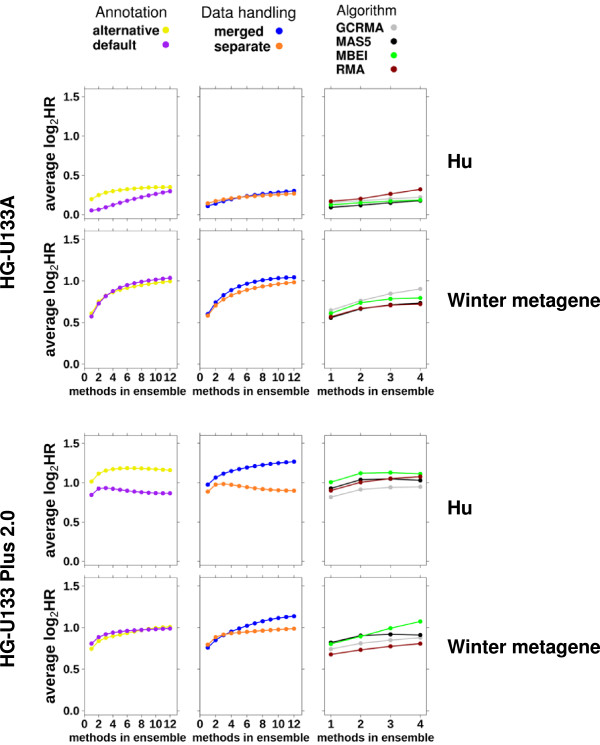
**Methods comparison.** Compare the contribution of annotation, dataset handling and algorithm choice as a function of the number of pre-processing methods included in the ensemble classification for the Hu signature and Winter metagene. Each point represents the log_2_ of the average hazard ratio using the ensemble approach of all combinations of x pipelines for the specific factor specified.

On both platforms there was a significant difference between annotations. On HG-U133A, alternative annotation had higher hazard ratios (p = 2.61 × 10^−2^, paired t-test). In direct contrast, HG-U133 Plus 2.0 performed better with default annotation (p = 1.31 × 10^−3^, paired t-test). By contrast, the optimal pre-processing algorithm was similar in both platforms, with RMA and MBEI performing better than GCRMA and MAS5 (p = 3.23 × 10^−3^-3.53 × 10^−7^, paired t-test). RMA and MBEI showed similar results (p = 0.241, paired t-test) as did GCRMA and MAS5 (p = 0.074, paired t-test). Furthermore, we analyzed the effect of changing the number of variants in the ensemble when creating only ensembles from common pipeline variants (Figure 6). Once again, variant success is not necessarily consistent across signatures.

### Ensemble of signatures

To further filter out unreliable classifications we investigated combining the classifications from two signatures. The Buffa metagene and the Winter metagene performed best across our analyses. These two signatures share 24 genes (out of 52 for Buffa metagene, out of 102 for Winter metagene). Expansion of the ensemble classification to only classify patients that both signatures agreed on (intersect of patients classified by both signatures) improved risk stratification (the hazard ratio) compared to ensemble evaluations of both signatures (Additional file 12: Figure S7).

To complete the analysis and expand the number of patients classified, we also pooled the unanimous classifications (the union of both signatures, excluding patients that were classified in contrasting risk groups). This failed to improve risk stratification compared to ensemble evaluations of both signatures; however, prognostic performance was improved over all the signatures’ individual pre-processing methods. Further, many more patients were classified than with the basic ensemble technique (Additional file 12: Figure S7), suggesting that ensembles of signatures could be used to further remove noise or to increase the number of patients given confident molecular classifications.

## Discussion

The purpose of pre-processing is to remove “noise” from the data. However, since no method is perfect, each pre-processing pipeline removes a somewhat different aspect of the “noise”. Indeed, groups around the world have focused on identifying the “optimal” pre-processing technique for different types of data [37,38]. The principle of ensemble classification is that by combining pre-processing approaches we can select the parts of the data which are reliable across the multiple approaches. The central tendency of this pool of methods is thus predicted to lie closer to the “true” value, and thereby to provide a better biomarker.

Although different pre-processing methods may cause some variation in the analysis, pre-processing is expected to have a minor effect on the core experimental results and conclusions [38,39]. Our previous work has indicated this is not the case and pre-processing caused major outcome differences in non-small cell lung cancer [9]. Here we systematically extend and deepen these analyses to explore the variation caused by algorithmic diversity in pre-processing.

At the single gene level substantial differences in prognostic power were seen in univariate analysis. Therefore pre-processing is part of the reason different studies identify different biomarker genes. Many authors will use public data to show that a given gene is prognostic; however, essentially all genes (~75%) can meet that criterion, depending on which platform and pre-processing technique is used. Single genes did not appear to behave the same across pipelines demonstrating variation in classification results are expected and signatures are dependent on the pre-processing platform they were discovered on. The fluctuation in prognostic ability for each gene partially explains why we get different results for multi-gene signatures and why there is such difficulty validating biomarkers between research groups [40].

In combination with data showing a plurality of gene sets are associated with outcome in breast cancer and other cancers [41-43], the variation suggests that mining of public data for prognostic significance is very prone to over-fitting and multiple-testing concerns. Therefore robust, permutation-based approaches need to be developed [44].

In non-small cell lung cancer, Starmans *et al.*[9] showed one example of a permutation-based approach where an ensemble biomarker classifier improved survival separation between low risk and high risk patient groups. Here we extended this finding showing that the method replicates on two microarray platforms representing 10 separate datasets in breast cancer for a series of 15 biomarkers. Across both platforms, there was not a single pipeline that unfailingly outperformed all other pipelines; therefore, the ensemble classification provides a generalized approach to improve biomarkers, both in terms of performance and stability, without determining the actual optimal pre-processing pipeline.

Furthermore, in many of the cases, the ensemble classifications outperformed all single pre-processing methods. The ultimate value of the ensemble classifiers as a concept was demonstrated with the Buffa metagene and Winter metagene. For these two signatures, any ensemble classifier comprising at least nine pipelines on HG-U133A or 20 pipelines on HG-U133 Plus 2.0 arrays generated superior risk stratifications compared to all the classifiers that used only a single pre-processing pipeline. Consequentially, ensemble classifiers are most definitely beneficial and should be used.

The ensemble approach did not improve all biomarkers. Biomarkers with generally bad risk stratifications across pre-processing pipelines still showed poor performance when combined in an ensemble. The ensemble approach magnified the separation of risk groups rather than corrected for a poor initial biomarker. Therefore the ensemble approach can also be used as a metric to assess the quality of biomarkers, distinguishing between poor and good signatures. By the statistical significance and consistency in risk stratification improvement across the datasets, the Buffa and Winter metagenes are shown to be strong, consistent signatures. By the same metric, Seigneuric 0% early and Starmans cluster 1, 2, 3 appear to be poor signatures validating previous findings where these signatures did not show prognostic power [20].

A disadvantage of the ensemble classification is that a fraction of patients are not classified. Only patients with robust risk classification across pipelines in the ensemble are assigned to risk groups. Here, using only the statistically significant ensembles 16% to 80% of the patients were not classified. The signatures showing significance on both platforms tend to have a higher percentage of patients classified (36% to 80%) than the signature significant on only on platform (16% to 68%) and the signatures not significant on either platform (15% to 46%). Nevertheless a patient classified as unreliable with one signature may be robustly classified using a different signature. This was shown by intersecting the Buffa and Winter metagenes, which resulted in improved prognostic power compared to the single pre-processing pipelines and classified more patients than with the two ensembles individually. One might consider taking this into account in biomarker-developement by attempting to construct ensembles that minimize the fraction of unclassified patients in the training dataset, although unclassified patients could resort to standard clinical care.

An important note about our approach to using the ensemble classification is the diversity of the pre-processing methods. Our choice of signature evaluation meant that a log_2_-transform on the data does not create different classifications. For example, the multiple methods MAS5 and log_2_ MAS5 pre-processing are actually only one pipeline variant and aren't filtering out additional unreliable patients. Here, the array of 24 pre-processing methods in reality only gives 16 unique classifications so there is less diversity than the numbers indicate. However, for other signatures, such as the risk-score and clustering methods evaluated previously [12], up to 24 unique classifications are possible.

In multiple signatures, the ensemble of all 24 pre-processing pipelines is an improvement but not the optimal ensemble classification (Additional file 8: Figure S5). So future analysis to refine the process and finesse which pipelines to use and how they should be combined would be advantageous. An important future direction is exploring how ensemble methods can be improved by incorporating greater algorithm diversity. Continual addition of diverse methods may increase the optimal ensemble classification or the optimal ensemble may be a certain combination of pipelines and new additions may not lead to an increase. In some cases, such as the Hu signature on HG-U133 Plus 2.0 addition of another separate data handling pipeline is not likely to increase the risk stratification but adding a merged data handling pipeline would be advantageous (Figure 6).

## Conclusions

We systematically show that differences in pre-processing create differences when using biomarkers. This effect of pre-processing is important for the research community to recognize and consider, as accurately accounting for it will advance biomarker discovery, validation and ultimately clinical application. We found that the Buffa metagene is the most consistent biomarker and therefore most clinical useful signature evaluated and we show that application of ensemble classification technique is beneficial for improving risk stratification both in terms of effect size and stability of biomarkers.

## Competing interests

All authors declare that they have no competing interests.

## Authors’ contributions

Database generation and curation: SH. Performed statistical and bioinformatics analyses: NSF, MHWS, PCB. Data interpretation: NSF, MHWS, PCB. Wrote the first draft of the manuscript: NSF. Initiated the project: PCB. Supervised research: MHWS, PL, PCB. All authors read and approved the final manuscript.

## Supplementary Material

Additional file 1: Table S1 Prognostic signature descriptions. Click here for file

Additional file 2: Table S2 Gene counts per prognostic signature. Click here for file

Additional file 3: Figure S1 Correlation of gene univariate analysis. Analysis of consistency between methods for the prognostic ability of each gene shown in Figure 2. The heatmap shows pairwise comparison of all the pipeline variants where the comparison is Spearman's correlation estimate of the FDR-adjusted p-values (q-values) for univariate Cox proportional hazard ratio modeling analysis of genes analyzed on the set of pipelines. Click here for file

Additional file 4: Figure S2 Platform comparison by signature. Comparison of hazard ratios for the series of prognostic signatures on HG-U133A and HG-U133 Plus 2.0. Hazard ratios were derived from Cox proportional hazard ratio modeling. Each triangle represents the ensemble classifier's hazard ratio and the circles represent the individual pipeline variants. The 95% confidence interval is shown for each ensemble. For the individual pipeline variants, the 95% confidence intervals are shown in Additional file 5: Table S3. Click here for file

Additional file 5: Table S3 Hazard ratio 95% confidence intervals for classifications on the individual pipeline variants. Click here for file

Additional file 6: Figure S3 Risk stratification across classification pipelines and prognostic signatures with equal number of patients classified. Comparison of hazard ratios (measure of risk stratification) and corresponding p-values from Cox proportional hazard ratio modeling between ensemble classifications and individual classifications on a subset of patients with the highest and lowest signature scores on (A) HG-U133A platform, (B) HG-U133 Plus 2.0 platform. The hazard ratio is represented by the size and colour of the dot and the background shade represents the p-value. Click here for file

Additional file 7: Figure S4 Signature comparison. Analysis of consistency across both significant prognostic signatures and signatures that were not (compared to Figure 5 A and B which only should significant signatures). Heatmaps are shown for the pair-wise comparison (measured as percent agreement of patient classifications) of all the single pipeline classifications for the individual pre-processing methods (A) and the ensemble scores derived from these individual classifications per patient for each signature (B). In B, the signatures are ordered by the number of patients classified unanimously across all the pipeline variants. From left to right, the number of patients classified in the ensemble for each signature decreases. Click here for file

Additional file 8: Figure S5 Ensemble hazard ratio range. The range of hazard ratios for ensembles from different number of pipeline variants. The horizontal pink dashed line shows the highest hazard ratio of the individual methods; all the ensembles above the line are improvements on current pre-processing practice. The x-axis indicate the number of pipeline variants combined to create ensembles. The grey background shows the numbers of pipeline variants where all the ensembles created are superior to every single individual method. The hazard ratio, p-value and number of patients classified for each ensemble shown is provided in Additional file 11: Table S4 and Additional file 12: Table S5. Click here for file

Additional file 9: Table S4. The hazard ratios, p-values and number of patients classified for all of the classifications on HG-U133A in Additional file 8: Figure S5. To make the data easier to use, each signature is in a separate a tab delimited table/text file and files for each platform are packaged and compressed separately. Each row in the tables is a patient classification and there are repeated rows since we were sampling the pipelines with replacement. The first 24 columns of each table is whether the pipeline specified in the column name is used in the ensemble classification with 1 meaning the pipeline is in the ensemble and 0 meaning it is not. Columns 25 – 27 are the hazard ratio, p-value and the number of patients classified for the classification respectively. Click here for file

Additional file 10: Table S5. The hazard ratios, p-values and number of patients classified for all of the classifications on HG-U133 Plus 2.0 in Additional file 8: Figure S5. To make the data easier to use, each signature is in a separate a tab delimited table/text file and files for each platform are packaged and compressed separately. Each row in the tables is a patient classification and there are repeated rows since we were sampling the pipelines with replacement. The first 24 columns of each table is whether the pipeline specified in the column name is used in the ensemble classification with 1 meaning the pipeline is in the ensemble and 0 meaning it is not. Columns 25 – 27 are the hazard ratio, p-value and the number of patients classified for the classification respectively. Click here for file

Additional file 11: Figure S6. Method correlation effect on hazard ratio. Comparison of the effect of method diversity in ensembles of 2 on the increase in hazard ratio from the maximum of the individual classifications for Winter metagene classifications on HG-U133A (on the left in pink) and HG-U133 Plus 2.0 (shown on the right in blue). Part A measures how correlated methods are by their percent agreement between methods (shown in Figure 5A) which is also equivalent to the number of patients classified. Part B measures the relatedness of the methods by the Spearman's correlation of how prognostic each gene is for a method (Additional file 3: Figure S1). Click here for file

Additional file 12: Figure S7. Combining signatures. Prognostic ability of combining the ensemble approach for the Winter metagene and the Buffa metagene was evaluated with Kaplan-Meier survival analyses. Hazard ratios and p-values are from Cox proportional hazard ratio modeling. The intersect is using only patients that are in agreement between Winter metagene and Buffa metagene. The union is pooling the patients from Winter metagene and Buffa metagene (excluding patients with conflicting risk classifications between the two signatures). Click here for file
