# Supplementary material for: Ensemble analyses improve signatures of tumour hypoxia and reveal inter-platform differences
Source: BMC Bioinformatics. 2014 Jun 6;15:170. doi: 10.1186/1471-2105-15-170 (PMC4061774; doi:10.1186/1471-2105-15-170)
Supplement: Additional file 1: Table S1 — Prognostic signature descriptions. [file 1471-2105-15-170-S1.pdf]

Supplementary Table 1 – Prognostic signature descriptions

Description of each hypoxia prognostic signature used.

Signature	Citation	# of genes	Development Description
Buffa metagene	[9]	51	Omnibus searched for gene expression studies/data sets in cancer, 8 were selected, Winter et al. Was used as a training data set<br>Tissue: frozen material extracted before chemotherapy, radiotherapy or adjuvant treatment<br>Microarray: Affymetrix U133A, B and plus2<br>Normalization: GCRMA
Chi	[10]	145	Cell lines (4): Human coronary artery endothelial cells, smooth muscle cells, human mammalian epithelial cells, and renal proximal tubule epithelial cells<br>Hypoxic conditions: 2% and 0.02% oxygen<br>Microarray: human cDNA microarrays containing 42,000 elements representing 27,291 unique genes, scanned with Axon Scanner 4000<br>Normalization: normalized to the ambient air control<br>harvested at the same time
Elvidge	[11]	178	Cell lines: MCF7 breast cancer and Hep3B hepatoblastoma<br>Hypoxic conditions: 1% oxygen<br>Microarray: HG-U133A GeneChips, HG-U133 plus 2 GeneChips<br>Normalization: GCRMA
Hu	[12]	13	Tissue: breast tumor<br>Microarray: Agilent Human oligonucleotide microarrays scanned on an Axon GenePix 4000B<br>Normalization: Lowess
Seigneuric 0% early	[13]	68	Used Data from Chi et al.<br>Cell lines (4): Human coronary artery endothelial cells, smooth muscle cells, human mammalian epithelial cells, and renal proximal tubule epithelial cells<br>Hypoxic conditions: 0.02% oxygen<br>Microarray: Affymetrix Human Genome U133A Array, Affymetrix Human Genome U133B Array<br>Normalization:
Seigneuric 2% early	[13]	34	Used Data from Chi et al.<br>Cell lines (4): Human coronary artery endothelial cells, smooth muscle cells, human mammalian

			epithelial cells, and renal proximal tubule epithelial cells<br>Hypoxic conditions: 2% oxygen<br>Microarray: Affymetrix Human Genome U133A Array, Affymetrix Human Genome U133B Array<br>Normalization:
Sorensen	[14]	28	Cell line: human uterine cervix squamous cell carcinoma<br>Hypoxic conditions: 5%, 1%, 0.1%, 0.01% and 0% oxygen,<br>Microarray: Human Genome U133 Plus 2.0 Array<br>Normalization: MAS 5.0
Winter metagene	[15]	101	Tissue: untreated head and neck squamous cell carcinomas from surgery<br>Microarray: Affymetrix U133plus2 GeneChips<br>Normalization: GCRMA
Cluster 1	[16]	69	Cell culture: Exponentially growing prostate (DU145), colon (HT29) and breast (MCF7) carcinoma cell<br>Hypoxic conditions: 0.0% oxygen<br>Microarray: Affymetrix HG-U133 Plus 2.0 GeneChips<br>Normalization: RMA
Cluster 2	[16]	246	Cell culture: Exponentially growing prostate (DU145), colon (HT29) and breast (MCF7) carcinoma cell<br>Hypoxic conditions: 0.0% oxygen<br>Microarray: Affymetrix HG-U133 Plus 2.0 GeneChips<br>Normalization: RMA
Cluster 3	[16]	157	Cell culture: Exponentially growing prostate (DU145), colon (HT29) and breast (MCF7) carcinoma cell<br>Hypoxic conditions: 0.0% oxygen<br>Microarray: Affymetrix HG-U133 Plus 2.0 GeneChips<br>Normalization: RMA
Cluster 4	[16]	95	Cell culture: Exponentially growing prostate (DU145), colon (HT29) and breast (MCF7) carcinoma cell<br>Hypoxic conditions: 0.0% oxygen<br>Microarray: Affymetrix HG-U133 Plus 2.0 GeneChips<br>Normalization: RMA
Cluster 5	[16]	162	Cell culture: Exponentially growing prostate (DU145), colon (HT29) and breast (MCF7) carcinoma cell

			Hypoxic conditions: 0.0% oxygen<br>Microarray: Affymetrix HG-U133 Plus 2.0 GeneChips<br>Normalization: RMA
Cluster 6	[16]	14	Cell culture: Exponentially growing prostate (DU145), colon (HT29) and breast (MCF7) carcinoma cell<br>Hypoxic conditions: 0.0% oxygen<br>Microarray: Affymetrix HG-U133 Plus 2.0 GeneChips<br>Normalization: RMA
Cluster 7	[16]	28	Cell culture: Exponentially growing prostate (DU145), colon (HT29) and breast (MCF7) carcinoma cell<br>Hypoxic conditions: 0.0% oxygen<br>Microarray: Affymetrix HG-U133 Plus 2.0 GeneChips<br>Normalization: RMA
