# Supplementary material for: Ensemble analyses improve signatures of tumour hypoxia and reveal inter-platform differences
Source: BMC Bioinformatics. 2014 Jun 6;15:170. doi: 10.1186/1471-2105-15-170 (PMC4061774; doi:10.1186/1471-2105-15-170)
Supplement: Additional file 2: Table S2 — Gene counts per prognostic signature. [file 1471-2105-15-170-S2.pdf]

Supplementary Table 2 - Gene counts per prognostic signature

The number of genes with data in each annotation and platform for the signatures.

Signature	# of genes	Default Annotation		Alternative Annotation	
		U133A	U133 Plus 2.0	U133A	U133 Plus 2.0
Buffa metagene	**51**	42	46	44	48
Chi	**145**	113	129	118	137
Elvidge	**178**	167	170	169	170
Hu	**13**	12	12	12	12
Seigneuric 0% early	**68**	54	67	52	65
Seigneuric 0% early	**34**	28	30	27	31
Sorensen	**28**	19	22	21	27
Winter metagene	**101**	79	90	77	92
Cluster 1	**69**	48	60	52	68
Cluster 2	**246**	205	231	211	246
Cluster 3	**157**	114	144	115	155
Cluster 4	**95**	61	86	64	92
Cluster 5	**162**	119	142	123	159
Cluster 6	**14**	8	11	10	14
Cluster 7	**28**	19	25	15	26
