# Supplementary figures and images for: Ensemble analyses improve signatures of tumour hypoxia and reveal inter-platform differences
Source: BMC Bioinformatics. 2014 Jun 6;15:170. doi: 10.1186/1471-2105-15-170 (PMC4061774; doi:10.1186/1471-2105-15-170)

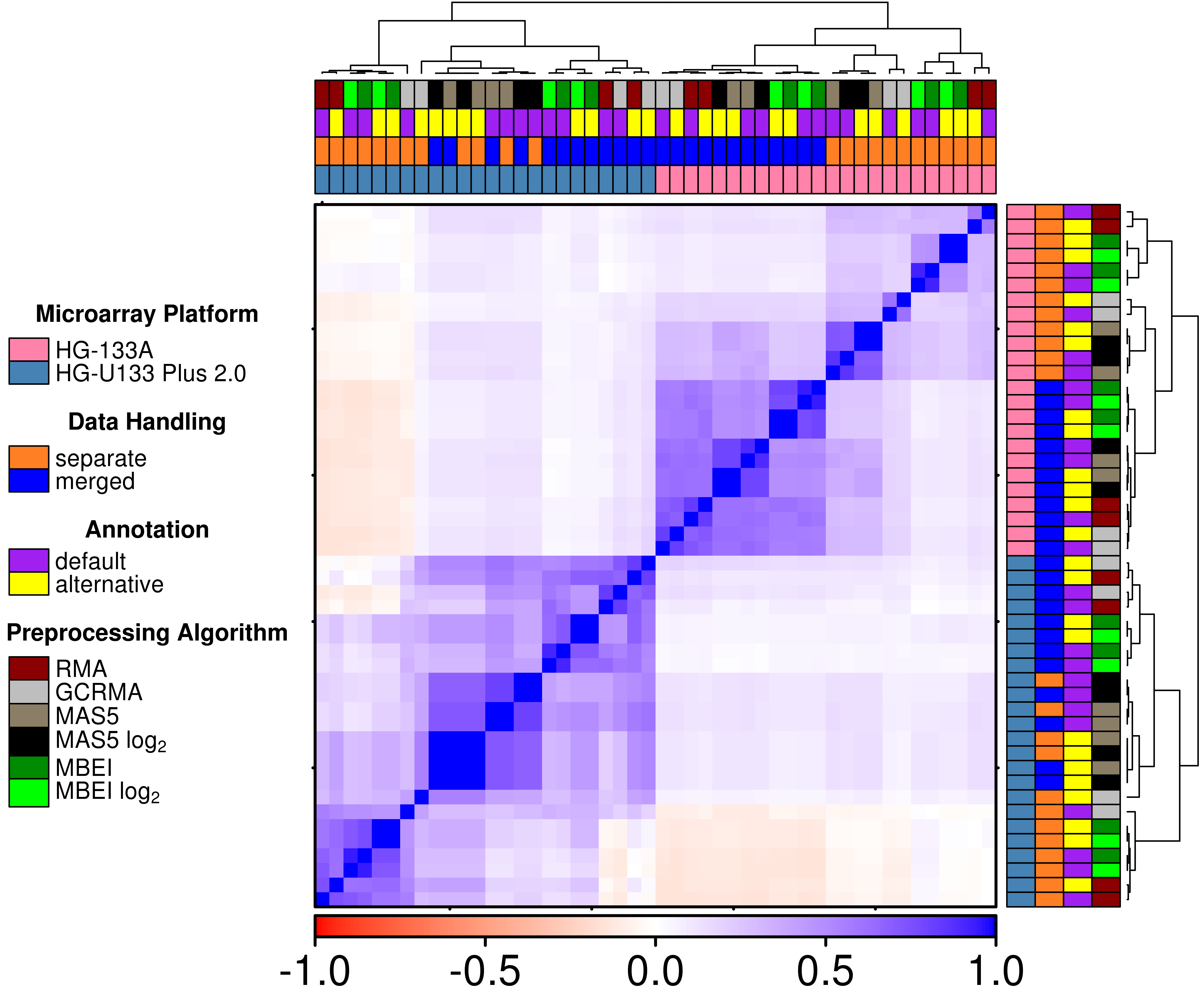

Supplement: Additional file 3: Figure S1 — Correlation of gene univariate analysis. Analysis of consistency between methods for the prognostic ability of each gene shown in Figure 2. The heatmap shows pairwise comparison of all the pipeline variants where the comparison is Spearman's correlation estimate of the FDR-adjusted p-values (q-values) for univariate Cox proportional hazard ratio modeling analysis of genes analyzed on the set of pipelines. [file 1471-2105-15-170-S3.tiff]

### Data Handling

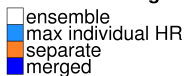

### Annotation

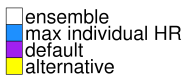

### Preprocessing Algorithm

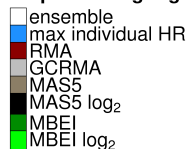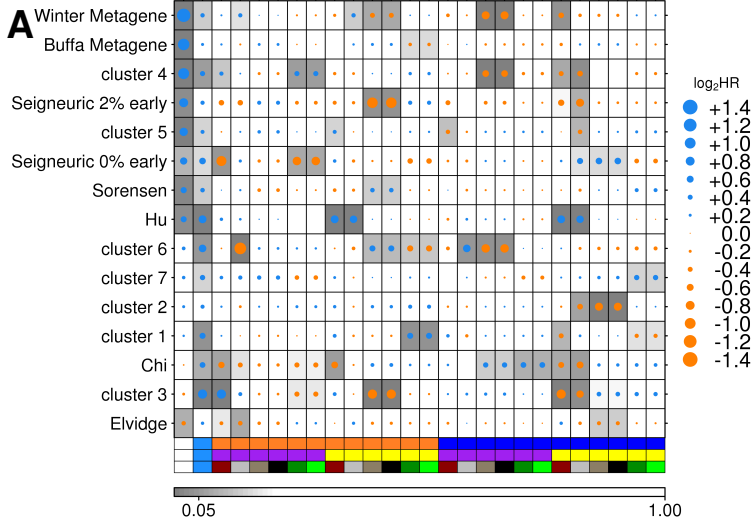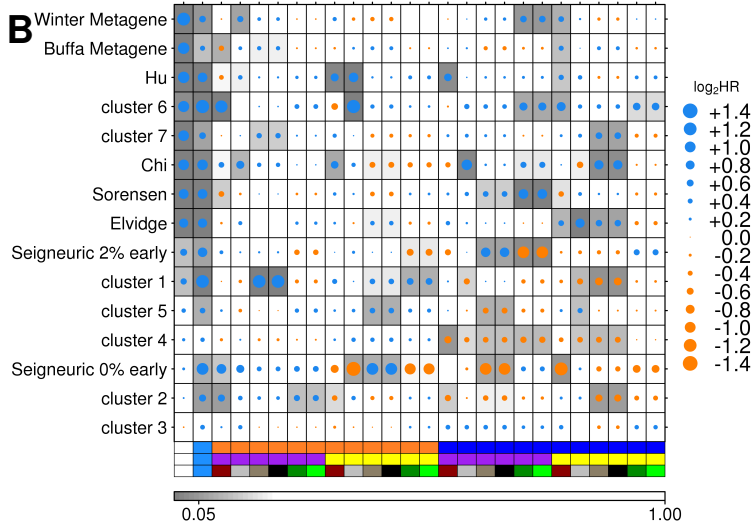

Supplement: Additional file 6: Figure S3 — Risk stratification across classification pipelines and prognostic signatures with equal number of patients classified. Comparison of hazard ratios (measure of risk stratification) and corresponding p-values from Cox proportional hazard ratio modeling between ensemble classifications and individual classifications on a subset of patients with the highest and lowest signature scores on (A) HG-U133A platform, (B) HG-U133 Plus 2.0 platform. The hazard ratio is represented by the size and colour of the dot and the background shade represents the p-value. [file 1471-2105-15-170-S6.pdf]

HG-U133A

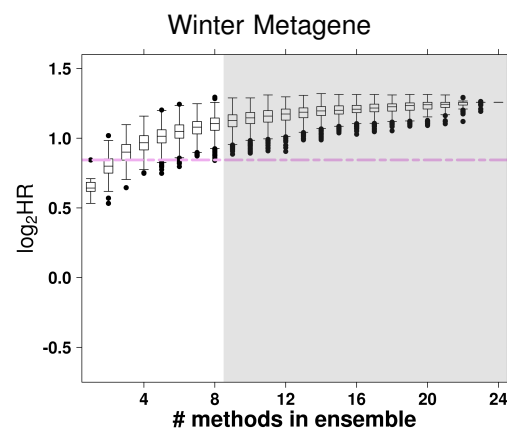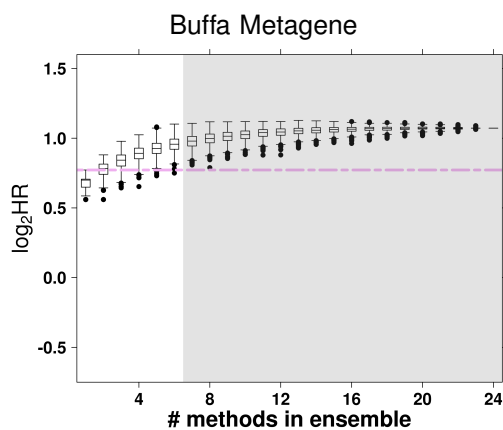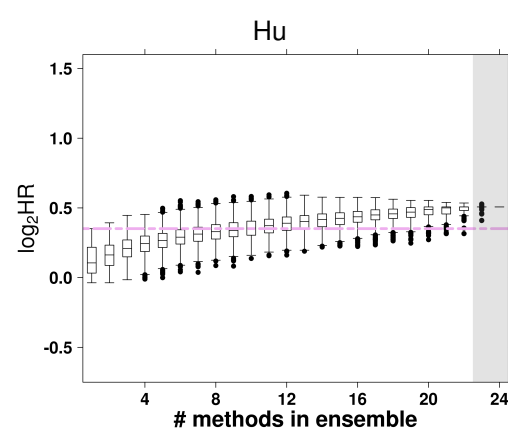

HG-U133 Plus 2.0

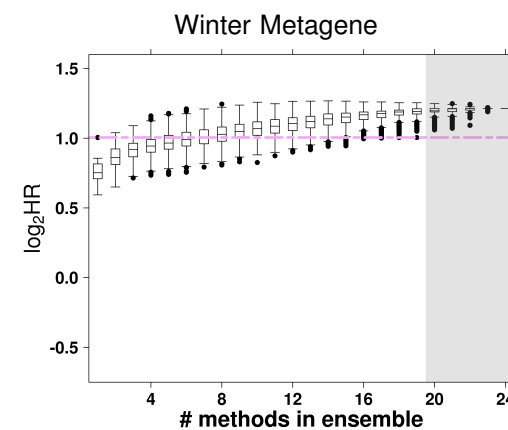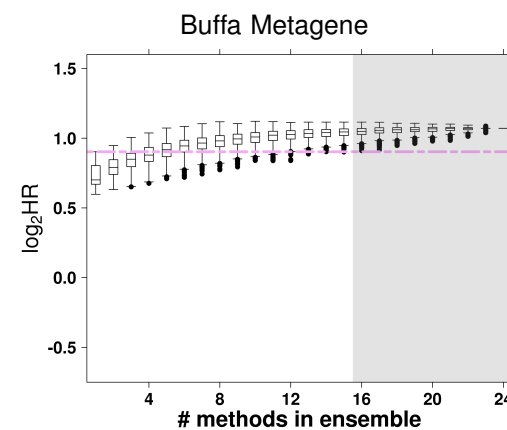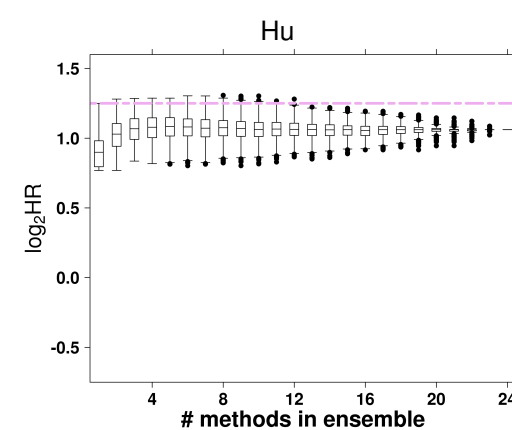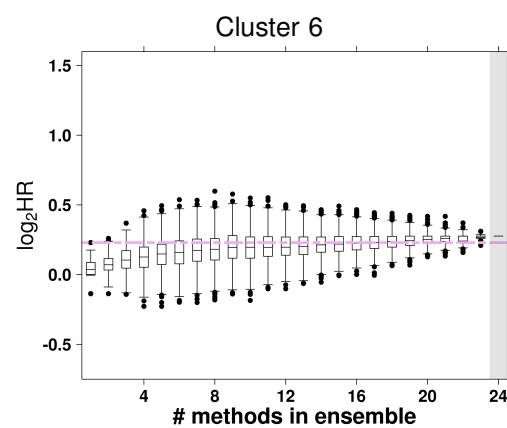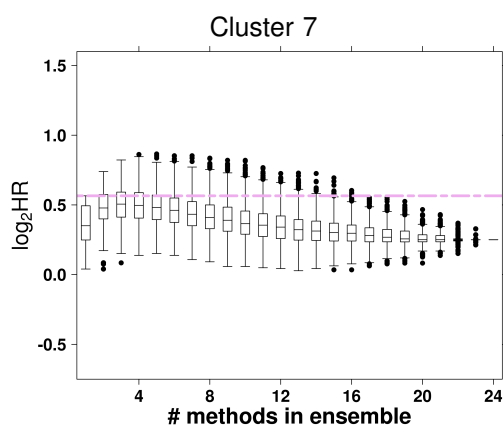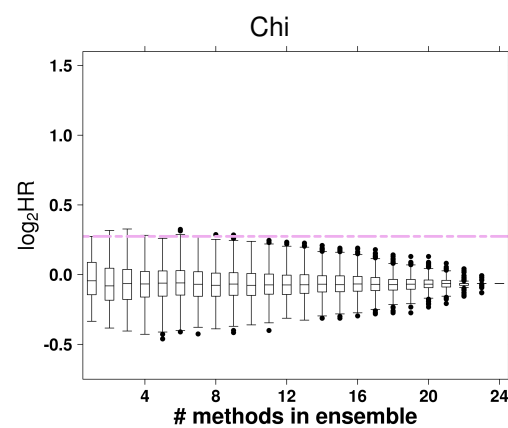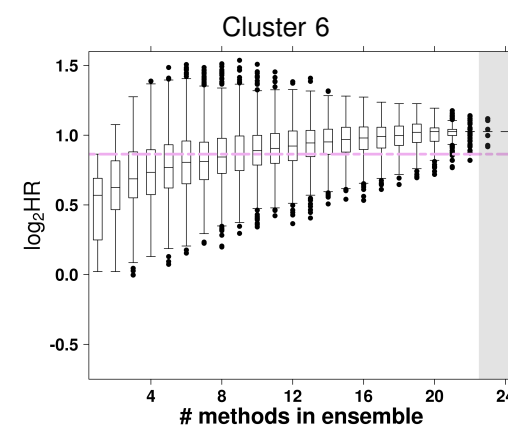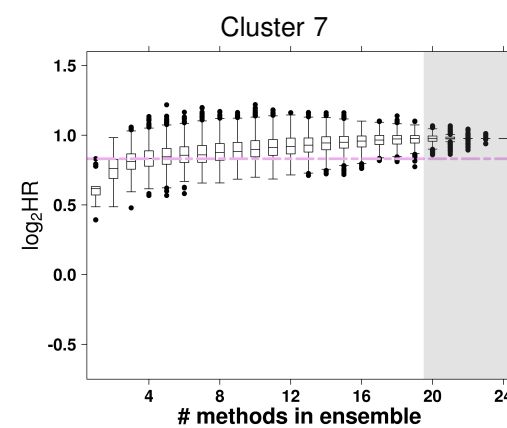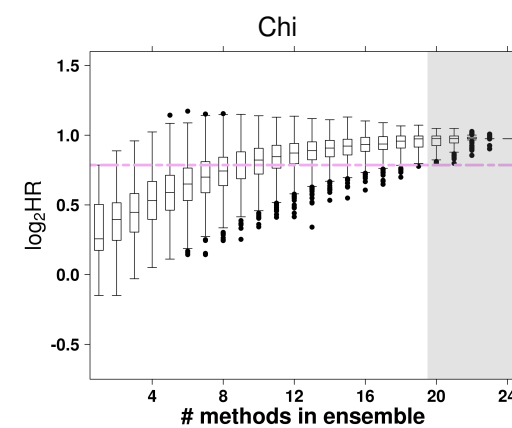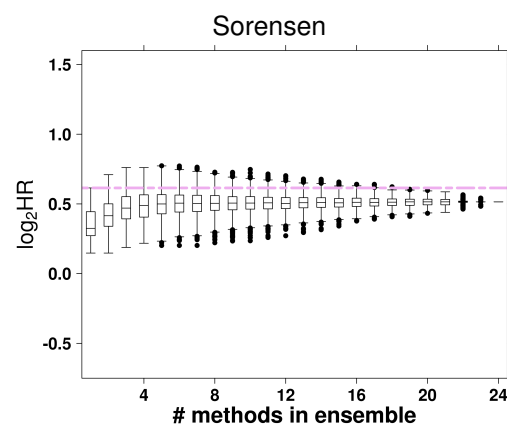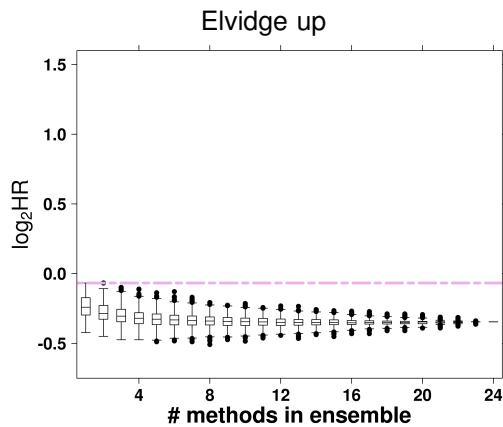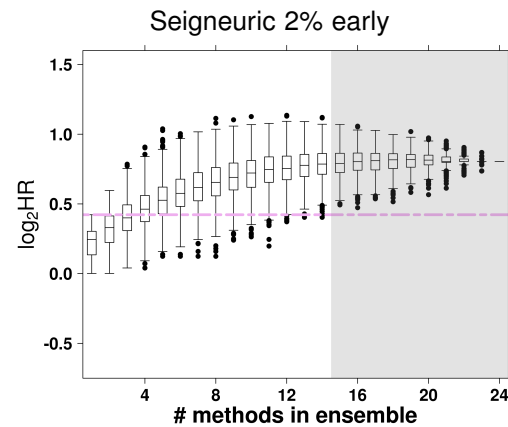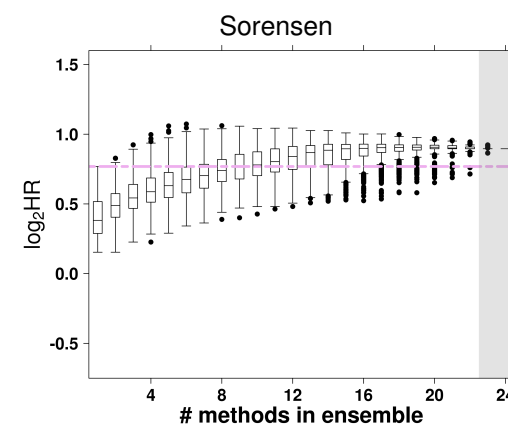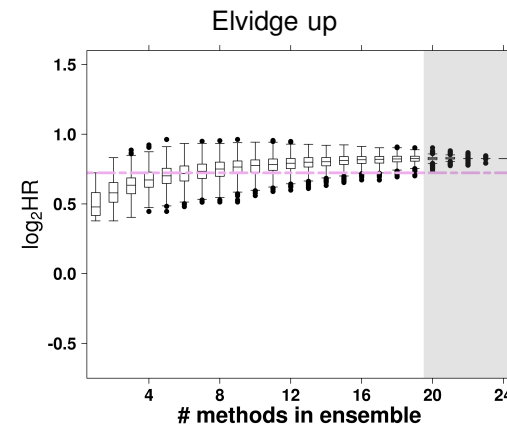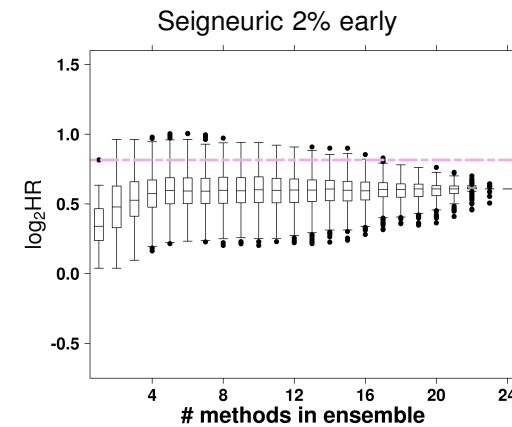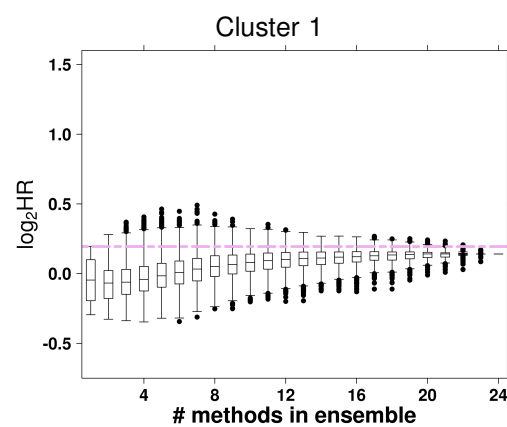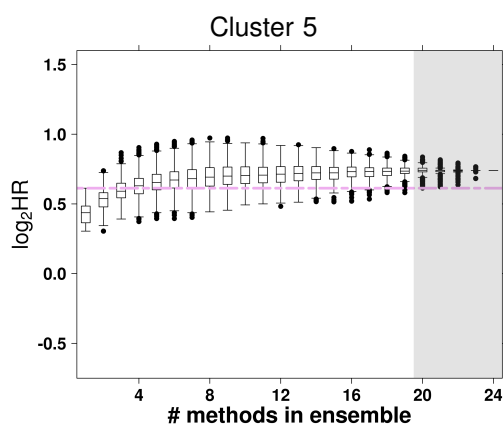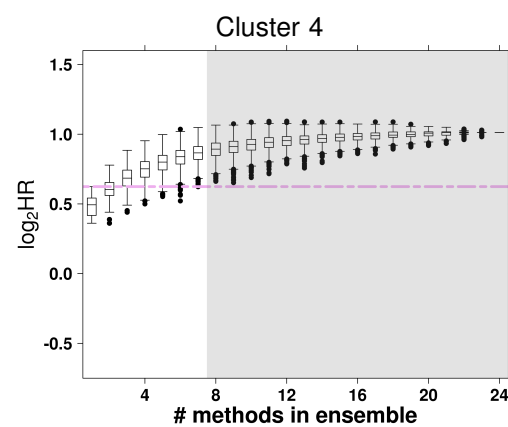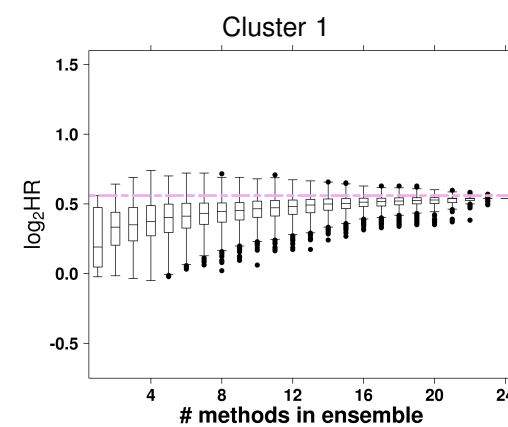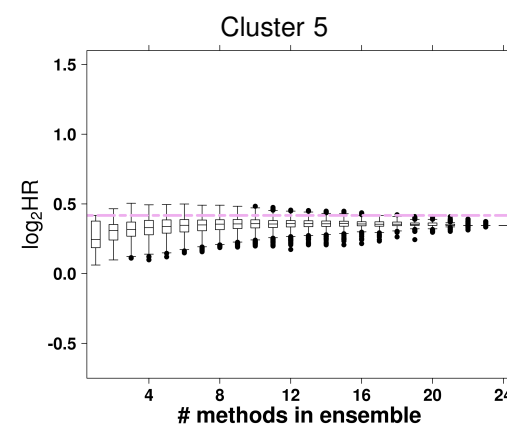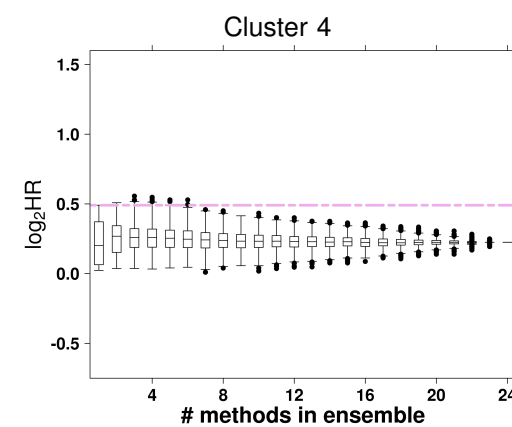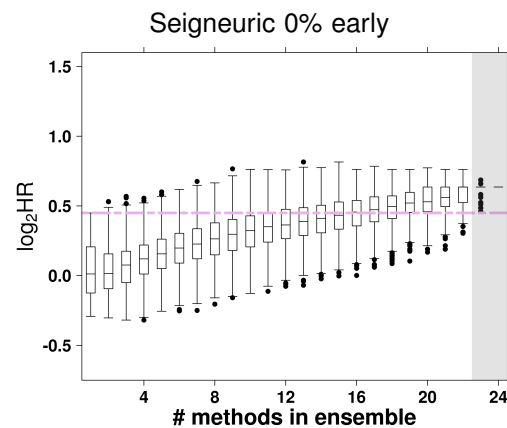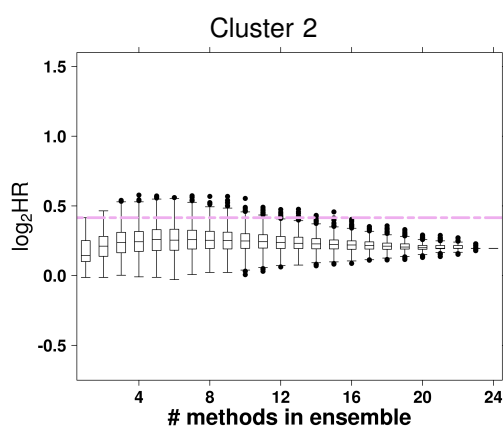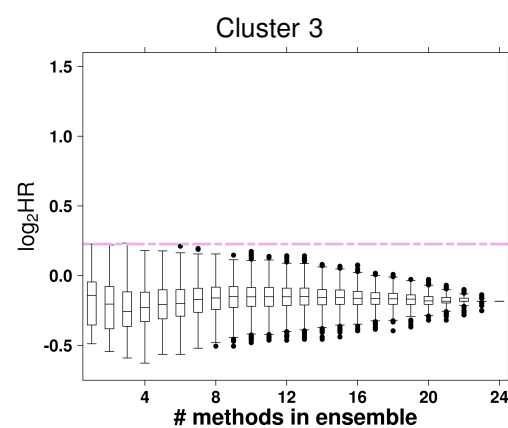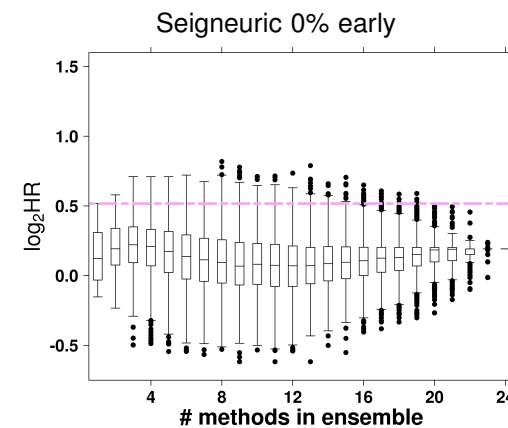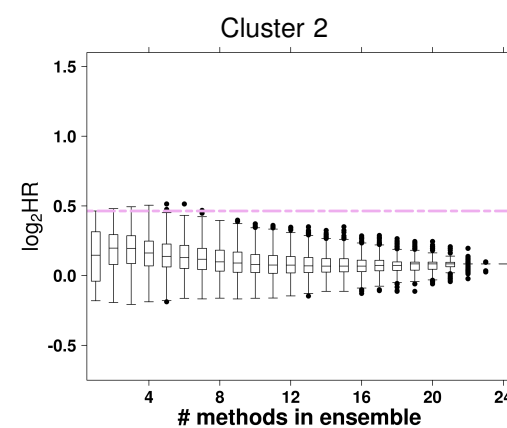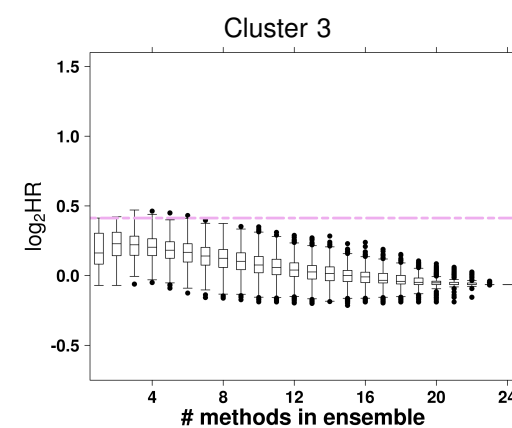

Supplement: Additional file 8: Figure S5 — Ensemble hazard ratio range. The range of hazard ratios for ensembles from different number of pipeline variants. The horizontal pink dashed line shows the highest hazard ratio of the individual methods; all the ensembles above the line are improvements on current pre-processing practice. The x-axis indicate the number of pipeline variants combined to create ensembles. The grey background shows the numbers of pipeline variants where all the ensembles created are superior to every single individual method. The hazard ratio, p-value and number of patients classified for each ensemble shown is provided in Additional file 11: Table S4 and Additional file 12: Table S5. [file 1471-2105-15-170-S8.pdf]

HG-U133A

Buffa Metagene

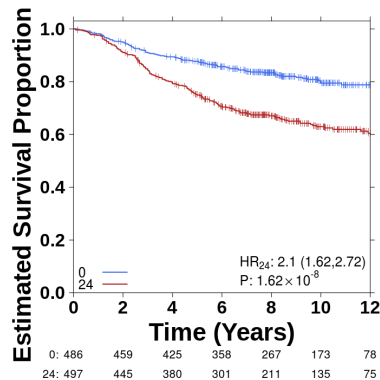

Winter Metagene

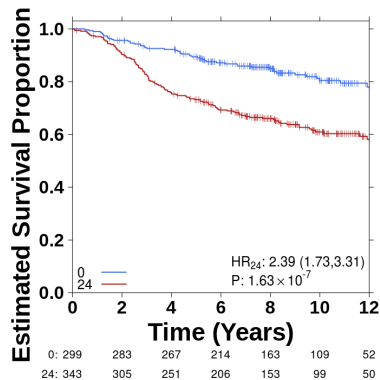

Intersect

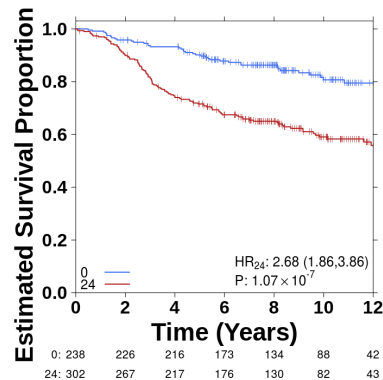

Union

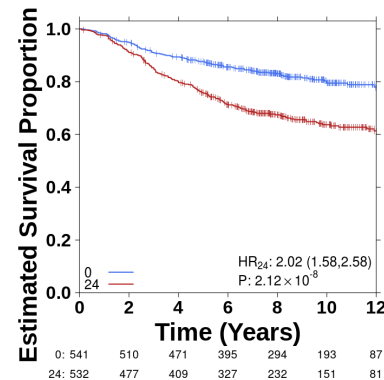

HG-U133 Plus 2.0

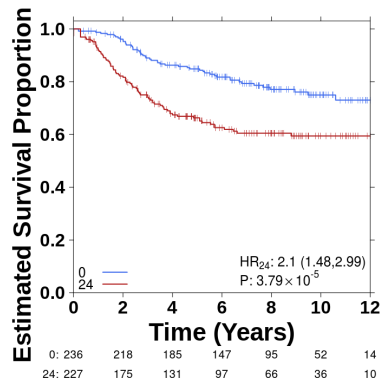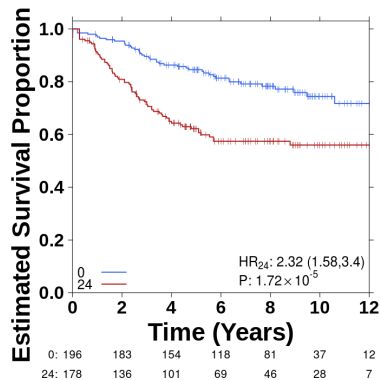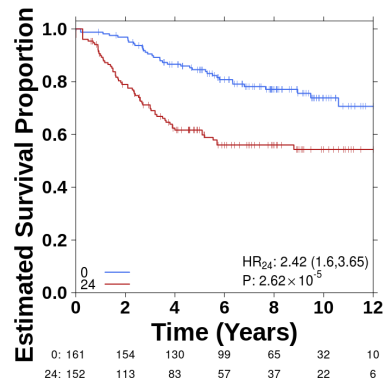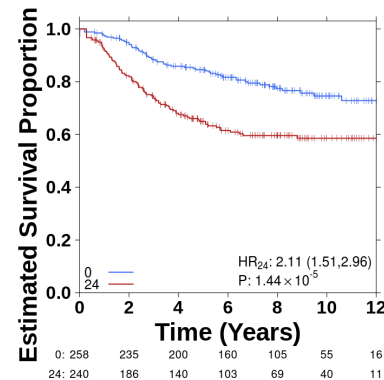

Supplement: Additional file 12: Figure S7. — Combining signatures. Prognostic ability of combining the ensemble approach for the Winter metagene and the Buffa metagene was evaluated with Kaplan-Meier survival analyses. Hazard ratios and p-values are from Cox proportional hazard ratio modeling. The intersect is using only patients that are in agreement between Winter metagene and Buffa metagene. The union is pooling the patients from Winter metagene and Buffa metagene (excluding patients with conflicting risk classifications between the two signatures). [file 1471-2105-15-170-S12.pdf]
