# Supplementary material for: Ensemble analyses improve signatures of tumour hypoxia and reveal inter-platform differences
Source: BMC Bioinformatics. 2014 Jun 6;15:170. doi: 10.1186/1471-2105-15-170 (PMC4061774; doi:10.1186/1471-2105-15-170)
Supplement: Additional file 4: Figure S2 — Platform comparison by signature. Comparison of hazard ratios for the series of prognostic signatures on HG-U133A and HG-U133 Plus 2.0. Hazard ratios were derived from Cox proportional hazard ratio modeling. Each triangle represents the ensemble classifier's hazard ratio and the circles represent the individual pipeline variants. The 95% confidence interval is shown for each ensemble. For the individual pipeline variants, the 95% confidence intervals are shown in Additional file 5: Table S3. [file 1471-2105-15-170-S4.pdf]

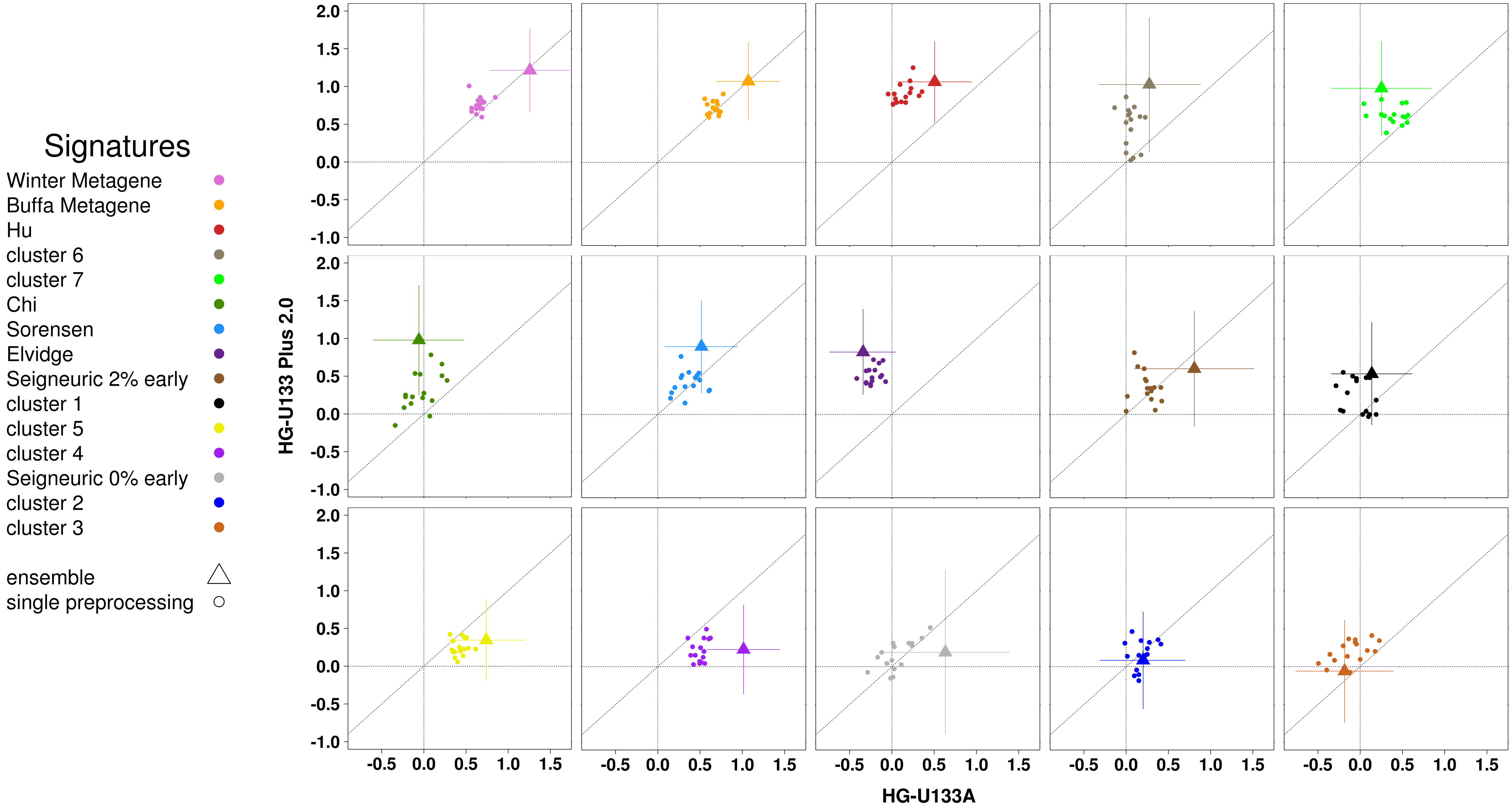

Signatures
Winter Metagene
Buffa Metagene
Hu
cluster 6
cluster 7
Chi
Sorensen
Elvidge
Seigneuric 2% early
cluster 1
cluster 5
cluster 4
Seigneuric 0% early
cluster 2
cluster 3
ensemble
single preprocessing
HG-U133 Plus 2.0
HG-U133A
2.0
1.5
1.0
0.5
0.0
-0.5
-1.0
-0.5
0.0
0.5
1.0
1.5
