# Supplementary material for: Ensemble analyses improve signatures of tumour hypoxia and reveal inter-platform differences
Source: BMC Bioinformatics. 2014 Jun 6;15:170. doi: 10.1186/1471-2105-15-170 (PMC4061774; doi:10.1186/1471-2105-15-170)
Supplement: Additional file 7: Figure S4 — Signature comparison. Analysis of consistency across both significant prognostic signatures and signatures that were not (compared to Figure 5 A and B which only should significant signatures). Heatmaps are shown for the pair-wise comparison (measured as percent agreement of patient classifications) of all the single pipeline classifications for the individual pre-processing methods (A) and the ensemble scores derived from these individual classifications per patient for each signature (B). In B, the signatures are ordered by the number of patients classified unanimously across all the pipeline variants. From left to right, the number of patients classified in the ensemble for each signature decreases. [file 1471-2105-15-170-S7.pdf]

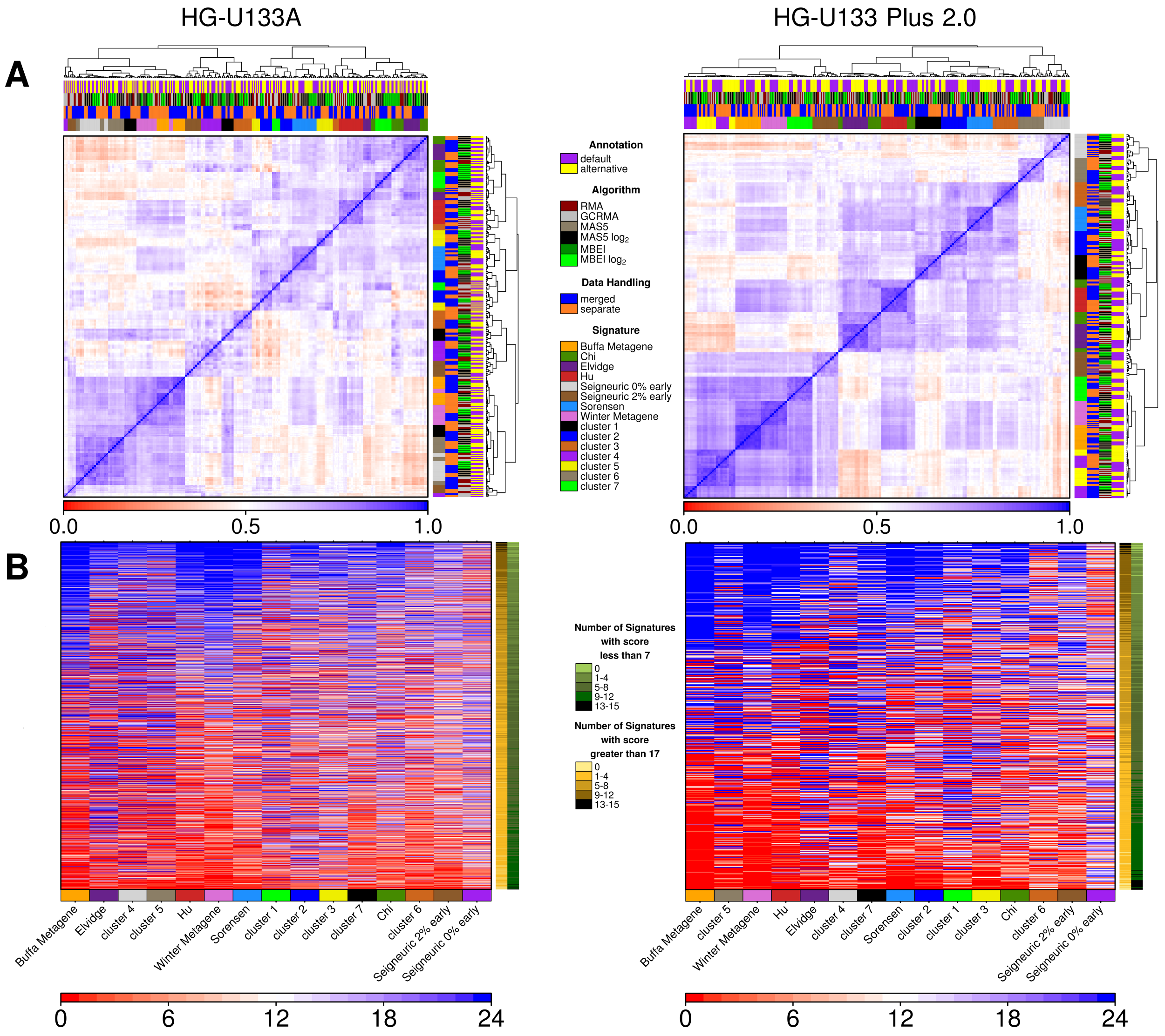
HG-U133A
HG-U133 Plus 2.0
A
B
Annotation
default
alternative
Algorithm
RMA
GCRMA
MAS5
MAS5 log2
MBEI
MBEI log2
Data Handling
merged
separate
Signature
Buffa Metagene
Chi
Elvidge
Hu
Seigneuric 0% early
Seigneuric 2% early
Sorensen
Winter Metagene
cluster 1
cluster 2
cluster 3
cluster 4
cluster 5
cluster 6
cluster 7
0.0
0.5
1.0
Number of Signatures with score less than 7
0
1-4
5-8
9-12
13-15
Number of Signatures with score greater than 17
0
1-4
5-8
9-12
13-15
Buffa Metagene
Elvidge
cluster 4
cluster 5
Hu
Winter Metagene
Sorensen
cluster 1
cluster 2
cluster 3
cluster 7
Chi
cluster 6
Seigneuric 2% early
Seigneuric 0% early
Buffa Metagene
cluster 5
Winter Metagene
Hu
Elvidge
cluster 4
cluster 7
Sorensen
cluster 2
cluster 1
cluster 3
Chi
cluster 6
Seigneuric 2% early
Seigneuric 0% early
0
6
12
18
24
