# Supplementary material for: Ensemble analyses improve signatures of tumour hypoxia and reveal inter-platform differences
Source: BMC Bioinformatics. 2014 Jun 6;15:170. doi: 10.1186/1471-2105-15-170 (PMC4061774; doi:10.1186/1471-2105-15-170)
Supplement: Additional file 11: Figure S6. — Method correlation effect on hazard ratio. Comparison of the effect of method diversity in ensembles of 2 on the increase in hazard ratio from the maximum of the individual classifications for Winter metagene classifications on HG-U133A (on the left in pink) and HG-U133 Plus 2.0 (shown on the right in blue). Part A measures how correlated methods are by their percent agreement between methods (shown in Figure 5A) which is also equivalent to the number of patients classified. Part B measures the relatedness of the methods by the Spearman's correlation of how prognostic each gene is for a method (Additional file 3: Figure S1). [file 1471-2105-15-170-S11.pdf]

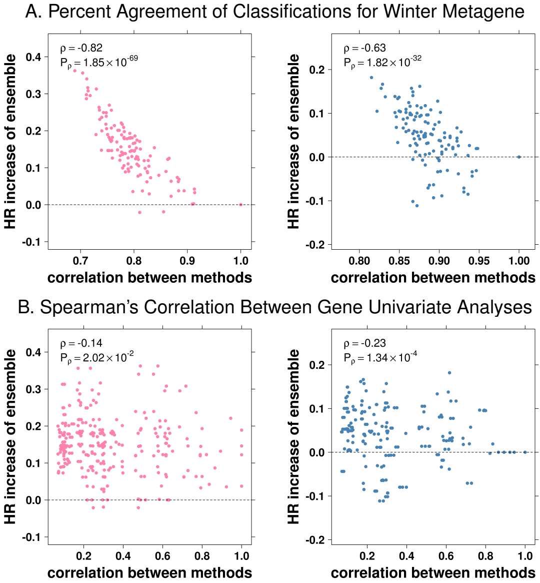
A. Percent Agreement of Classifications for Winter Metagene
ρ = -0.82
$P_\rho = 1.85 \times 10^{-69}$
ρ = -0.63
$P_\rho = 1.82 \times 10^{-32}$
HR increase of ensemble
correlation between methods
B. Spearman's Correlation Between Gene Univariate Analyses
ρ = -0.14
$P_\rho = 2.02 \times 10^{-2}$
ρ = -0.23
$P_\rho = 1.34 \times 10^{-4}$
HR increase of ensemble
correlation between methods
